# Pneumonia

**Published:** 1890-08

**Authors:** E. H. M. Parham

**Affiliations:** Fordyce, Ark.


### PNEUMONIA.

BY E. H. M. PARHAM, M. D., FORDYCE, ARK.

In the June number of the *Medical and Surgical Reporter*, page 696, will be found an article written by my friend, Dr. J. Knox Hodge, of Princeton, Ark, advocating the use of ergot, in the treatment of pneumonia.

While I acknowledge that the theory advanced by him would seem plausible in some degree, I cannot endorse him in placing my chief reliance in ergot in the treatment of inflammatory diseases, to the exclusion of what I believe to be more reliable remedies, to combat inflammation, located in any organ, whether it be the lungs, liver, spleen or brain; according to his own theory.

The form he uses is as follows, viz.:

℞. Fluid Ext. Ergot - - - - f℥ iv.

Tinct. Gelsemium.

Antmonial wine - - - - aa f℥ ii.

Mix. Sig. 30 drops every two hours, to be increased if necessary.

Now, it will be readily seen that he has combined two of the most potent remedies we have, in the treatment of local inflammation with the ergot, and gives the entire credit to ergot for his wonderful success. It reminds me of what occured when I first came to this State. An esteemed neighbor came to my house and found me with a chill. By way of encouragement, he remarked that I need not care about that, and said that Black Root (Leptandra) would cure me at once, and that he had a chill a short time before and it cured him.

I asked the old gentleman if he used no other remedy to prevent a return of the chill?

Yes, he said, there was some old quinine which had lost its strength and he used that, but of course the black root did all the good that was done.

Now, I know that Dr. J. K. Hodge is a too well posted physician not to find some other old remedy which may be reported to have lost its strength, such as calomel and Dover's powder, and bring them to the support, in this Southern climate, of his vaunted remedy (ergot) in time for the good of his patients.

Dr. Hodge says that he lost no cases of pneumonia last winter, while using his so-called ergot treatment, and I am pleased to say, that I, too, treated quite a number of cases of pneumonia, last winter and spring, without losing one, and I certainly did not use one drop of that unreliable and nauseous medicine (ergot). The chief remedies I used, are as follows:

℞. Calomel - - - - grs. x.
Dover's powders - - - grs. xx.

Mix and divide into six powders and take one every four hours with laxatives sufficient to prevent salivation.

I alternate the above with the following:

℞. Tinct. Gelsemium.
Tinct. Digitalis - - - aa fʒ i.
Sweet Spts. Nitre - - - f℥ ii.

Mix. Sig. Teaspoonful as above directed.

I also use counter-irritants freely. Time and space both forbid my treating fully the effects of ergot, but I will say this, that I have been using it nearly fifty years, whenever I found it indicated, and must say that I found it seldom indeed attended with good results in arresting hemorrhage or in contracting the non-striated muscular fibers, so as to reduce an engorged and enlarged viscus to its normal size and condition.

I am aware that I offer nothing new here, but I watch my patients closely, reduce the temperature when required, using such remedies as are least calculated to produce collapse, (a condition that I always dread) and when threatened, will ward off, if possible, with stimulants.

I have written the above in order that the readers of Dr. Hodge's ingeniously written article may be guarded against participating in his over-zeal in the treatment of pneumonia in favor of ergot and thereby lose valuable time in using what I conceive to be more reliable and therefore more useful remedies.

